# Toward a systems understanding of plant–microbe interactions

**DOI:** 10.3389/fpls.2014.00423

**Published:** 2014-08-25

**Authors:** Akira Mine, Masanao Sato, Kenichi Tsuda

**Affiliations:** ^1^Department of Plant Microbe Interactions, Max Planck Institute for Plant Breeding ResearchCologne, Germany; ^2^Okazaki Institute for Integrative Bioscience, National Institute for Basic Biology, National Institutes of Natural SciencesOkazaki, Japan

**Keywords:** systems biology, experimental network reconstruction, plant immunity, phytohormone, robustness, tunability, pathogen effector, microbial community

## Abstract

Plants are closely associated with microorganisms including pathogens and mutualists that influence plant fitness. Molecular genetic approaches have uncovered a number of signaling components from both plants and microbes and their mode of actions. However, signaling pathways are highly interconnected and influenced by diverse sets of environmental factors. Therefore, it is important to have systems views in order to understand the true nature of plant–microbe interactions. Indeed, systems biology approaches have revealed previously overlooked or misinterpreted properties of the plant immune signaling network. Experimental reconstruction of biological networks using exhaustive combinatorial perturbations is particularly powerful to elucidate network structure and properties and relationships among network components. Recent advances in metagenomics of microbial communities associated with plants further point to the importance of systems approaches and open a research area of microbial community reconstruction. In this review, we highlight the importance of a systems understanding of plant–microbe interactions, with a special emphasis on reconstruction strategies.

## INTRODUCTION

Systems biology is an area of biology that aims at a comprehensive and mechanistic understanding of complex and dynamic biological processes and phenomena. Systems biology approaches begin with system identification, where the (almost) whole components of a biological system are identified through functional and comparative genomics. This is followed by or coupled with systems analysis, where activities of individual components and their interactions are measured, and mathematical and computational models are built to describe and predict relationships of the system’s components and to explain intrinsic properties of the system. A generated model is used to build new hypotheses to be experimentally verified, and the experimentally verified information increases resolution of a next round of modeling. These repeated processes would advance a mechanistic understanding of how properties and traits of the biological system emerge (systems understanding), and eventually provide a basis for controlling the biological system and designing new biological traits (Kitano, 2002; Ukai and Ueda, 2010).

Due to immobile lifestyles, plants need to optimize their fitness within their living environments. Pathogenic and mutualistic microbes are major factors that influence plant fitness and use host plants for proliferation. Consistent with this, host plants and microbes have coevolved and acquired a number of mechanisms that modulate outcomes of their interactions (Jones and Dangl, 2006; Oldroyd, 2013). In addition, plant responses to microbes are affected by diverse abiotic environmental factors such as temperature and light (Hua, 2013). Thus, plant–microbe interactions are very complex and dynamic biological processes. Therefore, systems biology approaches are required to understand the true nature of plant–microbe interactions.

Plants rely on their innate immune system to resist pathogenic microorganisms. Pattern-triggered immunity (PTI) and effector-triggered immunity (ETI) are two defined modes of plant innate immunity against microbial pathogens (Jones and Dangl, 2006; Tsuda and Katagiri, 2010). PTI is triggered via recognition of conserved microbial molecules known as microbe-associated molecular patterns (MAMPs) by plasma membrane-localized pattern recognition receptors (PRRs; Boller and Felix, 2009; Monaghan and Zipfel, 2012). Well-characterized *Arabidopsis* PRRs include the flagellin sensing 2 (FLS2) for flg22 (a 22 amino acid peptide from the bacterial protein flagellin), the elongation factor-Tu (EF-Tu) receptor (EFR) for elf18 (a 18 amino acid peptide from the bacterial protein EF-Tu), and the chitin elicitor receptor kinase 1 (CERK1) for chitin (a part of fungal cell walls; Chinchilla et al., 2006; Zipfel et al., 2006; Miya et al., 2007; Wan et al., 2008). PTI is effective against most cases of microbial invasions. However, virulent pathogens have acquired diverse mechanisms to suppress PTI during coevolution (Jones and Dangl, 2006; Boller and He, 2009). For example, a set of effector proteins are delivered into plant cells to manipulate PTI signaling (Dou and Zhou, 2012). To counteract pathogen virulence, plants have evolved resistance (R) proteins, which are often nucleotide-binding leucine-rich repeat proteins (NLRs) as intracellular receptors that specifically recognize pathogen effectors directly or indirectly and that in turn activate ETI (Jones and Dangl, 2006; Jacob et al., 2013). For instance, *Arabidopsis* NLRs, resistance to *Pseudomonas syringae* 2 (RPS2) and resistance to *P. syringae* pv. *maculicola* 1 (RPM1), recognize actions of the bacterial effectors AvrRpt2 and AvrRpm1, respectively (Jones and Dangl, 2006).

Salicylic acid (SA), jasmonic acid (JA), and ethylene (ET) are immune-related phytohormones. It is generally accepted that SA signaling plays a major role in immunity against biotrophs and hemibiotrophs which require living hosts for multiplication, such as *Hyaloperonospora arabidopsidis* (*Hpa*) and *P. syringae*, respectively (Glazebrook, 2005). In contrast, JA and ET signaling are major contributors of immunity against necrotrophs which actively kill hosts during infection (Glazebrook, 2005). JA, ET, and SA are produced in some cases of PTI and ETI (Spoel et al., 2003; Liu and Zhang, 2004; Mur et al., 2008; Tsuda et al., 2008, 2013; Halim et al., 2009; Tintor et al., 2013). Since JA, ET, and SA signaling pathways intimately interact with synergism and antagonism (Pieterse et al., 2009), these signaling pathways form a complex network to regulate plant immunity.

In this review, we describe how a systems understanding of plant–microbe interactions can be achieved using functional genomics such as protein–protein interactomics, transcriptomics, and metagenomics. We will then focus on experimental reconstruction of complex biological networks, such as the phytohormone signaling network, as a powerful approach to elucidate mechanisms underlying complex and dynamic properties of plant–microbe interactions.

### A PHYSICAL INTERACTION NETWORK BETWEEN PLANT PROTEINS AND PATHOGEN EFFECTORS

**FIGURE 1:**
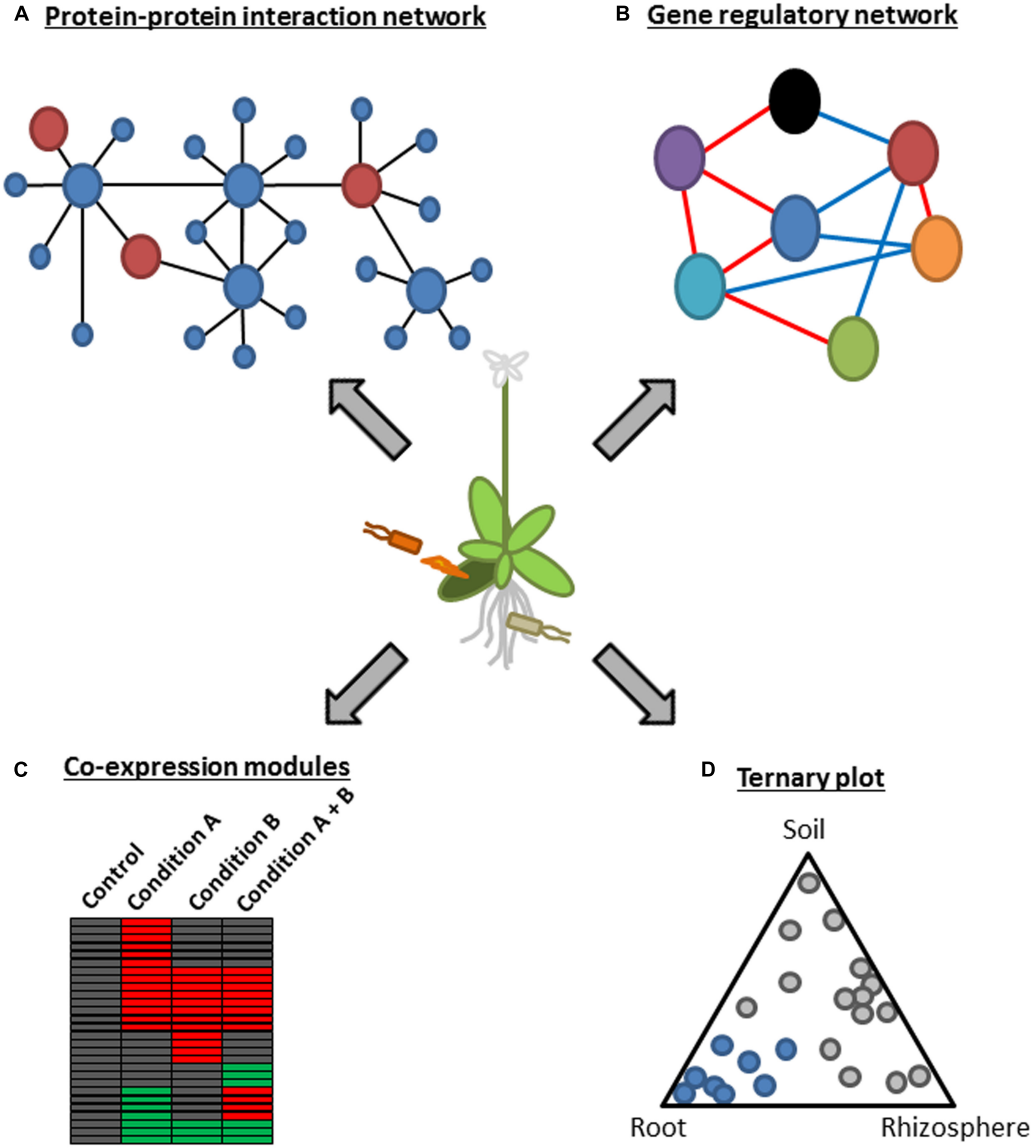
**A schematic representation of systems approaches using functional genomics. (A)** A protein–protein interaction network represents a global picture of a system at the protein level. In this example, several plant hub proteins (large blue circles) that interact with many plant proteins (small blue circles) are targeted by microbial effectors (large red circles). This approach was used in Mukhtar et al. (2011). **(B)** Gene regulatory network modeling infers regulatory relationships among components of a system. As an example, a network consisting of seven components (circles with different colors) with positive and negative regulatory relationships (red and blue lines, respectively) is depicted. This approach was used in Sato et al. (2010). **(C)** Co-expression module analysis is useful to visualize behavior of a system under certain conditions. As an example, co-regulated genes under different conditions are visualized in the heatmap. Red and green boxes indicate up-regulated and down-regulated genes in a certain condition, respectively. This approach was used in Zou et al. (2011), Atkinson et al. (2013), Prasch and Sonnewald (2013), and Rasmussen et al. (2013). **(D)** A ternary plot is used to show influence by three variables on composition. This example depicts bacterial OTUs whose relative abundance changes according to the three compartments, soil, rhizosphere, and root. Blue circles mark OTUs enriched in the root compartment. This approach was used in Bulgarelli et al. (2012) and Lundberg et al. (2012).

Microbial pathogens range from viruses, bacteria, oomycetes to fungi but often have common hosts. For example, the hemibiotrophic bacterium, *P. syringae,* and the obligate biotrophic oomycete, *Hpa*, can colonize the model plant *Arabidopsis*, raising a question whether there is a common mechanism employed by these evolutionally distant pathogens to suppress *Arabidopsis* immunity. This question was addressed by generating protein–protein interaction networks between *Arabidopsis* proteins and effectors from these pathogens based on genome-wide yeast two-hybrid analysis (**Figure 1A**; Mukhtar et al., 2011). This approach identified 165 putative effector targets, of which 18 were targeted by effectors from both pathogens. Most of the common effector targets were experimentally proved to be important for immunity by genetic analysis. Remarkably, they were enriched in proteins that have more than 50 interactors and were therefore considered as the hubs of a highly interconnected *Arabidopsis* protein–protein interaction network. For instance, one of such hub proteins, CSN5a, a component of COP9 signalosome, interacted with 29 distinct effectors from *Hpa* and *P. syringae* and was a negative regulator of immunity against *Hpa*. These results suggest that irrespective of their lifestyles, the two pathogens from different kingdoms have independently evolved effectors that converge onto cellular hub proteins to attack the plant immune system (Mukhtar et al., 2011). This is further supported by a study showing that most *Hpa* effectors promoted bacterial growth in *Arabidopsis* (Fabro et al., 2011). As similar observations were obtained in other systems such as plant–virus, human–virus, and human–bacteria interactions (Calderwood et al., 2007; De Chassey et al., 2008; Dyer et al., 2008; Elena and Rodrigo, 2012), targeting hub components of host immune systems is likely a common and effective strategy of distinct pathogens.

### NETWORK MODELING USING EXPRESSION PROFILES OF GENETIC MUTANTS

Transcriptome analysis in combination with genetics and a network modeling algorithm was applied to generate a signaling network during immunity against *P. syringae* carrying the effector AvrRpt2 (Sato et al., 2010). The modified version of locally linear embedding (LLE) enabled detection of weak regulatory relationships by subjecting the residual from the first round of LLE to another round of LLE (Sato et al., 2010). Regulatory relationships among 22 known immune signaling components representing different signaling sectors such as the MAP kinase (MAPK) sector (*MPK3* and *MPK6*; Liu and Zhang, 2004; Wang et al., 2007), the nitric oxide (NO) sectors (*NOA1* and *NIA2*; Wilkinson and Crawford, 1991; Guo et al., 2003) the reactive oxygen species (ROS) sector (*RBOHD* and *RBOHF*; Torres et al., 1998), the callose sector (*PMR4*; Nishimura et al., 2003) and phytohormones were modeled based on similarities in mRNA expression profiles of *Arabidopsis* mutants deficient in one of the network components (**Figure 1B**; Sato et al., 2010). The resulting static model inferred extensive negative regulatory relationships among signaling sectors during AvrRpt2-ETI. For example, the JA sector was negatively linked to most of other signaling sectors including the SA sector. The model also inferred a novel negative link between the SA sector and the early MAMP-triggered signaling sectors such as the MAPK, callose, and ET sectors. Importantly, this prediction was experimentally validated, as mutual inhibition between flg22- and SA-induced marker gene inductions was observed in a dose dependent manner, highlighting the significance of the modeling approach based on expression profiles of multiple genetic mutants to generate testable hypotheses (Sato et al., 2010). The negative regulatory relationships among the signaling sectors may make the ETI signaling network robust against perturbation by, for example, pathogen effectors, as perturbation of one sector can be compensated by the other sectors through a switch-like mechanism: one sector(s) gets activated due to the loss of negative effects from the other sector(s) perturbed by pathogens (Sato et al., 2010).

### INTEGRATION OF MULTIPLE INPUTS INTO GENE REGULATION

Environmental factors such as temperature and light are known to affect plant immunity (Hua, 2013), suggesting that plants integrate complex information coming from environments and microbes to optimize their response. Yet, how plants integrate complex information into response is not well understood. Several recent studies tackled this question by comparing transcriptional responses of *Arabidopsis* to single and multiple stresses (**Figure 1C**; Atkinson et al., 2013; Prasch and Sonnewald, 2013; Rasmussen et al., 2013). All these studies found that transcriptional responses to multiple stresses are not easily predictable from the responses to single stresses. For example, effects of multiple stresses on gene expression were not simply additive. Consistent with this unpredictability, transcriptional response to the viral pathogen, *turnip mosaic virus*, was significantly altered when combined with additional abiotic stresses, such as heat and drought (Prasch and Sonnewald, 2013). This was associated with enhanced viral susceptibility under these abiotic stress conditions. Moreover, transcriptional response to the parasitic nematode, *Heterodera schachtii,* in combination with drought stress was significantly different from those to the single stresses (Atkinson et al., 2013). *Rapid alkalinization factor-like 8*, *methionine gamma lyase,* and *azelaic acid induced 1* are genes that showed unpredictable expression patterns under the combined stress condition. Overexpression or loss of function of these genes resulted in altered responses to drought stress as well as to nematode infection. These results suggest that specific transcriptional responses to combinatorial stresses are crucial parts of stress tolerance mechanisms in plants.

It is an interesting question whether signal integration for gene regulation is achieved at a transcription factor which is regulated by multiple signaling pathways or at a gene promoter where multiple transcription factors recognize distinct *cis*-regulatory elements (CREs). Although a number of CREs have been experimentally identified in promoters of stress-responsive genes, this information is not sufficient to correctly predict stress-responsive gene expression. Based on co-expression patterns of *Arabidopsis* genes under many different biotic and abiotic stresses, a number of putative CREs with characteristics of authentic *cis-*elements were computationally identified (**Figure 1C**; Zou et al., 2011). Strikingly, prediction of gene up-regulation by salt, ultraviolet or flg22 was markedly improved by considering binary combinations of known or putative CREs compared to that based on the presence or absence of single CREs. Thus, stress-responsive gene expression is apparently governed by multiple CREs which are signal integration sites for gene regulation, and further systems analysis will be required to untangle the complexity.

### STRUCTURE IDENTIFICATION OF PLANT ROOT-INHABITING BACTERIAL MICROBIOTA

Plant–microbe interaction studies have been mostly focused on binary interactions, typically consisting of a single host plant and either pathogenic or mutualistic microbes (Jones and Dangl, 2006; Oldroyd, 2013). However, plants are surrounded by numerous microbes in natural environments, especially in soil, where one of the richest bacterial diversities exists (Gans et al., 2005). Recently, two independent research groups developed similar experimental pipelines for classifying bacterial 16S ribosomal DNA sequences into operational taxonomic unit (OTU) based on high-resolution pyrosequencing, and defined bacterial communities associated with *Arabidopsis* roots (Bulgarelli et al., 2012; Lundberg et al., 2012). In accordance with prior studies, plant-associated bacterial OTUs that are present inside or firmly attached to roots were significantly different from OTUs found in rhizosphere (defined as soil particles associated with roots) and in bulk soil (**Figure 1D**), suggesting a selection mechanism(s) that shapes the structure of root-inhabiting bacterial microbiota. Strikingly, by comparing bacterial community profiles associated with different *Arabidopsis* accessions and in soils from different locations, both studies independently identified a similar profile of root-inhabiting microbiota and reached the same conclusion that the soil type had a greater influence than the host genotype on the bacterial community composition (Bulgarelli et al., 2012; Lundberg et al., 2012). This suggests that the selection process may be facilitated by enrichment and/or exclusion of particular bacteria through interactions among different bacterial species present in a soil. Overall, these studies demonstrated the power of metagenomics for characterization of interactions between plants and microbial communities.

### A STEPWISE RECONSTRUCTION OF A PLANT IMMUNE SIGNALING NETWORK FROM A GROUND LEVEL STATE

**FIGURE 2:**
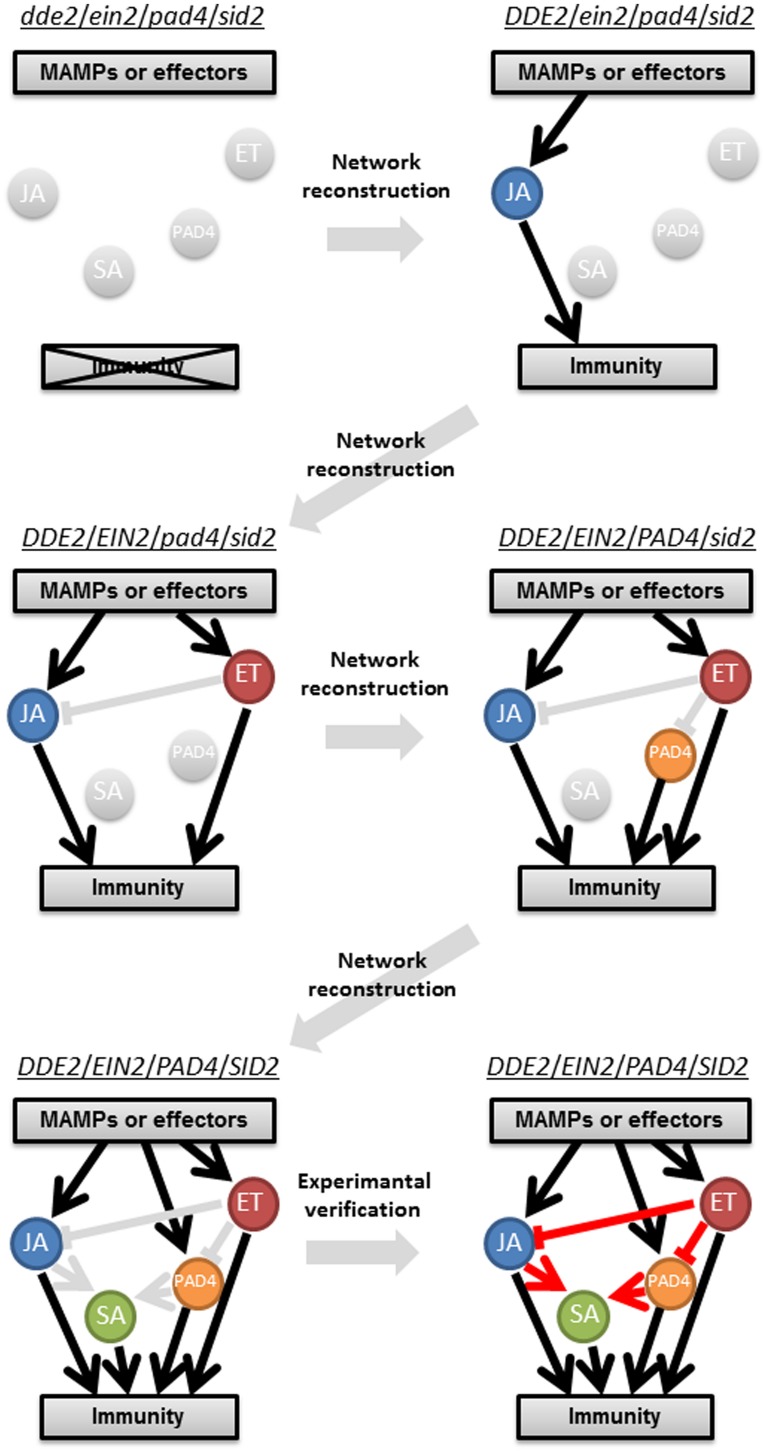
**A schematic representation of a stepwise reconstruction of a plant immune signaling network from a ground level state.** The *Arabidopsis dde2*/*ein2*/*pad4*/*sid2-*quadruple mutant is considered a ground level state of the plant immune signaling network consisting of the JA, ET, PAD4, and SA signaling sectors. Network reconstruction from the ground level state to the wild-type state via all combinatorial mutants is conducted to quantitatively measure contribution of the individual sectors to immunity (black lines) and activities (circles) and predict their regulatory relationships (gray lines). These predictions are experimentally verified (red lines). For simplicity, only one example is shown for triple, double, and single mutants. This approach was used in Tsuda et al. (2009) and Kim et al. (2014).

One of the longstanding questions in plant–pathogen interactions is why ETI response is very robust compared to PTI, which is overcome by virulent pathogens. A systems analysis using an *Arabidopsis* quadruple mutant and combinatorial mutants regarding the core immune signaling components, JA, ET, SA, and *phytoalexin deficient 4* (*PAD4*), revealed differential properties of PTI and ETI signaling networks, which provided an answer for this question (**Figure 2**; Tsuda et al., 2009). *Delayed-dehiscence 2 (DDE2)*, *ethylene insensitive 2 (EIN2),* and *salicylic acid induction-deficient 2 (SID2)* are essential components of JA, ET, and SA signaling, respectively (Alonso et al., 1999; Wildermuth et al., 2001; Park et al., 2002). *PAD4* is required for pathogen-induced SA accumulation and other immune responses (Jirage et al., 1999), and the latter is considered as the PAD4 signaling sector. Thus, the JA, ET, PAD4, and SA signaling sectors are all compromised in the *dde2/ein2/pad4/sid2*-quadruple mutant. Growth measurement of *P. syringae* showed that flg22-triggered immunity (flg22-PTI) and AvrRpt2-triggered immunity (AvrRpt2-ETI) are largely (∼80%) lost in the quadruple mutant. Hence, we consider the quadruple mutant as a (almost) ground level state of the plant immune signaling network accounting for flg22-PTI and AvrRpt2-ETI. The stepwise reconstruction (triple, double, and single mutants to the wild-type) of the plant immune signaling network from the ground level state (the quadruple mutant) followed by signaling allocation analysis revealed that contrary to previous ideas, the JA, ET, PAD4, and SA signaling sectors can all positively contribute to both flg22-PTI and AvrRpt2-ETI. The analysis also illustrated differential relationships among the signaling sectors in PTI and ETI: partly synergistic and partly compensatory in PTI but almost exclusively compensatory in ETI. For example, the interaction between the PAD4 and SA sectors is synergistic in PTI but compensatory in ETI. Thus, reconstructing a network from a ground level state is a powerful approach to understand the true contribution of a signaling sector in and properties of a highly interconnected network.

Although a synergistic interaction in PTI can be easily attenuated by a pathogen effector that disrupts one of the synergistically interacting signaling sectors, compensatory interactions such as in ETI would make the signaling network highly robust to perturbations. An interesting question is if robust immunity is correlated with robust gene expression. A genome-wide expression profile showed that SA is not essential during AvrRpt2-ETI for regulation of the majority of SA-responsive genes which are regulated in an SA-dependent manner in PTI (**Figure 1C**; Tsuda et al., 2013), demonstrating extensive compensation by other signaling mechanism(s) for the loss of SA. This seems reasonable because hemibiotrophic and biotrophic pathogens have diverse mechanisms to perturb SA signaling (Zheng et al., 2012; Caillaud et al., 2013; Jiang et al., 2013; Gimenez-Ibanez et al., 2014). The two immune-related MAPKs, MPK3, and MPK6, are activated with different kinetics during PTI and ETI: transient during flg22-PTI but sustained during AvrRpt2-ETI (Tsuda et al., 2013). The SA compensation in ETI seems to be regulated by, in part, sustained activation of MPK3 and MPK6. Thus, although the MAPKs are shared by PTI and ETI, different activation kinetics leads to different downstream events (Tsuda et al., 2013). ROS production and Ca^2+^ flux are also known to be more sustained during ETI than PTI (Shapiro and Zhang, 2001; Torres et al., 2006; Gao et al., 2013). Thus, time-resolved analysis is needed to fully understand downstream signaling mechanisms.

It should be noted that contribution of the four signaling sectors to immunity differs depending on trigger of immunity and/or pathogens (Tsuda et al., 2009; Maekawa et al., 2012). Therefore, further elucidation of network components is needed to understand the complete structure of the plant immune signaling network. MAPKs (Meng and Zhang, 2013; Tsuda et al., 2013), calcium-dependent protein kinases (Tena et al., 2011; Gao et al., 2013), NO (Zeidler et al., 2004; Zeier et al., 2004), ROS (Torres et al., 2002, 2006), and other phytohormones such as abscisic acid, auxin, and gibberellin (Asselbergh et al., 2008; Robert-Seilaniantz et al., 2011; Mang et al., 2012) are important players in plant immunity and should be integrated in further network analysis.

### A STEPWISE RECONSTRUCTION OF A PATHOGEN EFFECTOR REPERTOIRE

**FIGURE 3:**
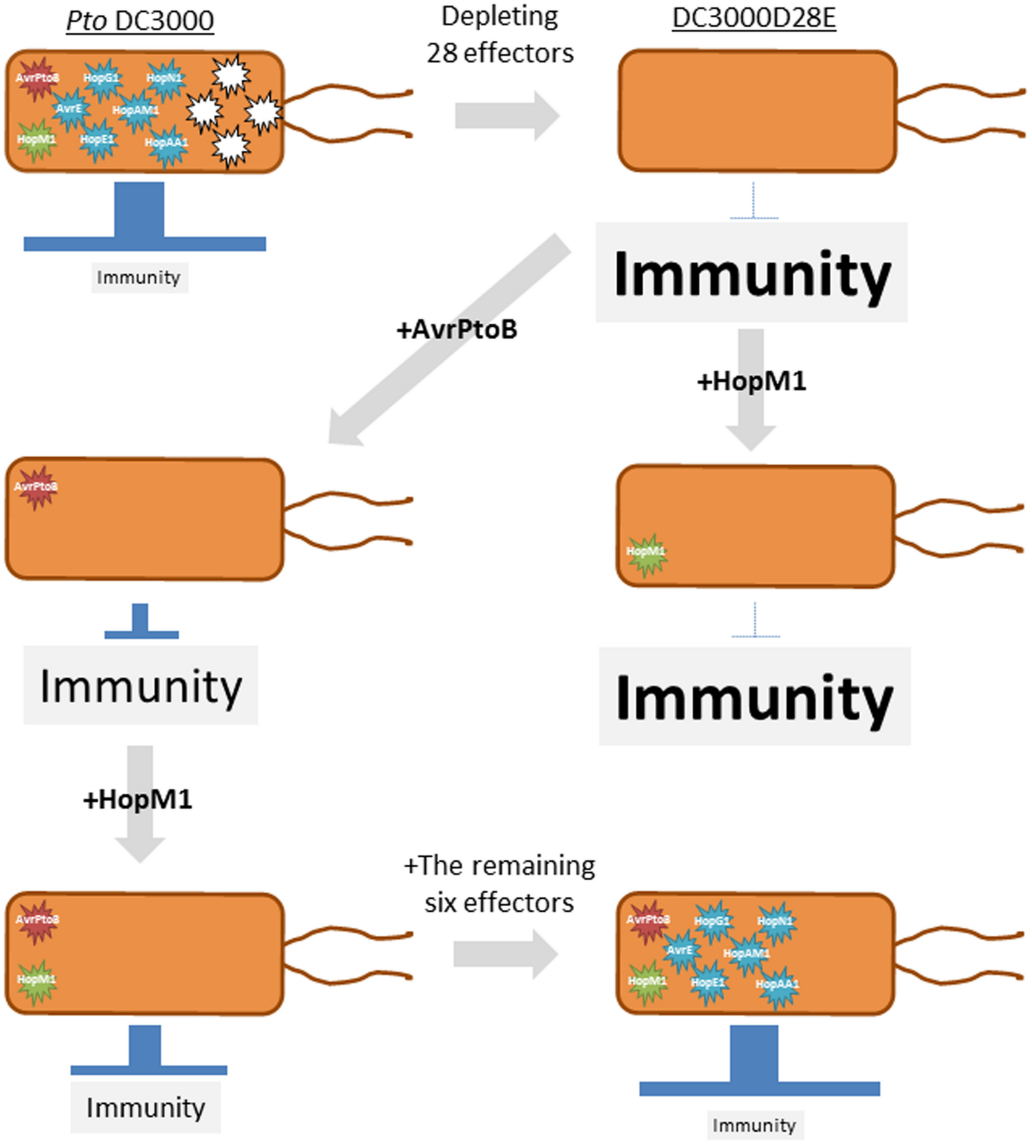
**A schematic representation of a stepwise reconstruction of a functional effector repertoire of *Pseudomonas syringae*.** The *P. syringae* pv. *tomato* (*Pto*) DC3000 mutant lacking 28 effectors (DC3000D28E) is considered a ground level state for virulence. AvrPtoB but not HopM1 is sufficient to promote *in planta* bacterial growth. HopM1 requires AvrPtoB to promote bacterial growth. The remaining six effectors (AvrE, HopE1, HopG1, HopAM1, HopAA1, and HopN1) support bacterial growth to near the wild-type level in the presence of AvrPtoB and HopM1. This approach was used in Cunnac et al. (2011).

Although individual functions of pathogen effectors have been studied, how these effectors function in a coordinated manner has been rarely investigated (Lindeberg et al., 2012). Therefore, a stepwise reconstruction of effectors was used to understand a pathogen virulence strategy (Cunnac et al., 2011). *P. syringae* pv. *tomato* DC3000 (*Pto*) is a model pathogen for systems analysis because virtually all effector candidates that are injected by the type III secretion system (T3SS) were identified (Lindeberg et al., 2006). The *Pto* mutant (DC3000D28E), which is deficient in the 28 effectors, was found to grow less than the T3SS-deficient mutant in *Nicotiana benthamiana.* Therefore, this functionally effector less mutant can be considered as a ground level state (Cunnac et al., 2011). A stepwise reconstruction of the effector repertoire to restore virulence of DC3000D28E identified a minimal set of eight effectors with hierarchical functions (AvrPtoB, HopM1, AvrE, HopE1, HopG1, HopAM1, HopAA1, and HopN1; **Figure 3**). AvrPtoB blocks initiation of PTI signaling by targeting PRRs such as FLS2 (Göhre et al., 2008; Gimenez-Ibanez et al., 2009) and the co-receptor BRI1-associated kinase 1 (BAK1; Shan et al., 2008; Monaghan and Zipfel, 2012). AvrPtoB alone was sufficient to promote growth of DC3000D28E (Cunnac et al., 2011). Bacterial growth was further promoted when HopM1, which targets a PTI component involved in vesicle trafficking (Nomura et al., 2006), was introduced together with AvrPtoB, suggesting that attenuation of receptor functions is a prerequisite for the function of HopM1 (Cunnac et al., 2011). The remaining six effectors supported bacterial growth to near the wild-type level when introduced into DC3000D28E harboring AvrPtoB and HopM1. HopE1, HopG1, HopAM1, HopAA1, and HopN1 seem less important for the bacterial virulence in *N. benthamiana,* because these effectors are lacking in the genome of *P. syringae* pv. *syringae* B728a and *P. syringae* pv. *tabaci* 11528, both of which cause disease in *N. benthamiana* (Vinatzer et al., 2006; Studholme et al., 2009), suggesting that these five effectors can be replaced with other effectors. It would be interesting to investigate whether these two *P. syringae* strains use effectors with functions similar to or distinct from the five effectors to promote bacterial growth. In summary, reconstruction of the functional effector repertoire from the ground level state together with the comparative genomics of *P. syringae* strains revealed the virulence strategy by which the bacterial pathogen manipulates plant immunity by deploying a few core effectors and many interchangeable effectors (Lindeberg et al., 2012).

### MODELING SIGNAL FLOWS USING EXPERIMENTAL NETWORK RECONSTRUCTION WITH MULTIPLE INPUTS AND OUTPUTS

The plant immune signaling network needs to be robust against pathogen attack and at the same time tunable to achieve optimal fitness since plants combat a diverse range of microbial pathogens including biotrophs and necrotrophs and since unnecessary immune responses have a negative impact on plant fitness (Tsuda and Katagiri, 2010; Alcázar et al., 2011; Mengiste, 2012). The PTI signaling network has some level of robust property although the level of robustness is lower compared to that in ETI (Tsuda et al., 2009). To elucidate the mechanisms underlying intrinsic properties of the PTI signaling network, such as robustness and tunability, the stepwise network reconstruction (Tsuda et al., 2009) was used to generate a dynamic model that describes signal flows and their contributions to immunity in the network consisting of the JA, ET, PAD4, and SA sectors (Kim et al., 2014). Expression levels of marker genes for each of the four sectors at two different time points and growth inhibition of two different *P. syringae* strains were measured as proxies of signaling sector activities and immune outputs, respectively, after the treatment with three different MAMPs, flg22, elf18 and a modified form of chitin, chitosan (Silipo et al., 2010) as well as mock treatment (**Figure 2**). Using this multifactorial quantitative data set, a highly predictable dynamic PTI network model was generated. We describe four key findings below.

First, the model predicted that the ET sector suppresses the JA and PAD4 sectors, which is a source of the robustness in the PTI signaling network. Second, although it is often thought that JA signaling inhibits SA signaling (Vlot et al., 2009), the JA sector was predicted to activate the SA sector. This prediction was surprising but experimentally verified as introducing the *dde2* mutation into the genotypes containing the *pad4* mutation abolished the flg22-induced SA accumulation to the same level as in *sid2*, indicating that the positive effect of the JA sector on the SA sector is evident only when the PAD4 sector is missing (Kim et al., 2014). This clearly points to the significance of combinatorial perturbations: simultaneous perturbations of multiple components are necessary to truly understand component’s functions in a highly interconnected network. Third, the model revealed that different MAMPs activate the four signaling sectors with different strength, resulting in different immune outputs. For example, flg22 strongly activates the JA, ET, and PAD4 sectors, which leads to strong contributions of the PAD4 and SA sectors to immunity against *P. syringae*. In contrast, elf18 and chitosan predominantly activate the JA and ET sectors, resulting in weaker immunity against the bacterial pathogens. Since chitosan is a MAMP from fungal pathogens including necrotrophs, it may have been selected to prioritize the JA and ET sectors over the other sectors to mount immunity effective against this type of pathogens (Glazebrook, 2005). Given that a microbe likely presents multiple MAMPs, it is tempting to speculate that plants tune sector activities of the PTI signaling network by sensing different compositions of MAMPs from different microbes to tailor appropriate immune responses for a specific condition. Last, the contribution of the PAD4 sector to immunity against the bacterial pathogens was clearly larger than that of the SA sector. Although a primary function of PAD4 is often thought to be the amplification of SA signaling (Vlot et al., 2009), this result suggests it may be the other way around: a major function of the SA sector is to activate the PAD4 sector rather than its direct contribution to immunity against biotrophic and hemibiotrophic pathogens. It will be necessary to characterize activities of the PAD4 and SA sectors independently of each other in order to re-define the relationship between the two sectors in plant immunity.

## CONCLUSION AND PERSPECTIVES

The first step of systems biology, system identification, has been achieved in many studies of plant–microbe interactions by means of functional and comparative genomics using model organisms. However, only some of these studies reached the second step, systems analysis, which is the most critical step to understand complex and dynamic properties of plant–microbe interactions (Tsuda et al., 2009, 2013; Sato et al., 2010; Cunnac et al., 2011; Mukhtar et al., 2011; Bulgarelli et al., 2012; Lundberg et al., 2012; Kim et al., 2014). Indeed, systems analysis using reconstruction approaches revealed how a plant immune signaling network is structured to be robust and tunable and how pathogen effectors manipulate plant immunity (Tsuda et al., 2009; Cunnac et al., 2011; Kim et al., 2014). Furthermore, reconstruction of microbial communities would undoubtedly facilitate our understanding of selection mechanisms and functions of plant root-inhabiting bacterial microbiota (Bulgarelli et al., 2012; Lundberg et al., 2012), as shown in studies of phyllosphere and gut microbiota (Atarashi et al., 2013; Bodenhausen et al., 2014). All these studies provide bases for improving future agricultural productivity and food security. For instance, we will, in principle, be able to develop chemical compounds that target core effectors to control pathogens effectively (Cunnac et al., 2011). Another idea will be potentiation of plant immunity by, for example, conferring ETI-like robustness to PTI (Tsuda et al., 2009, 2013; Kim et al., 2014). Notably, transgenic potato plants that trigger ETI-like sustained MAPK activation through recognition of MAMPs were generated (Yamamizo et al., 2006). This transgenic plants showed no developmental abnormalities but exhibited high resistance to both the necrotrophic fungus, *Alternaria solani,* and the biotrophic oomycete, *Phytophthora infestans* (Yamamizo et al., 2006). Conventional molecular genetic approaches for rigorous testing of hypotheses emerging from systems analysis will further elucidate molecular mechanisms underlying complexity and dynamics of plant–microbe interactions, which will again provide a basis for controlling the outcomes of plant–microbe interactions.

So far, most systems biology studies have utilized data sets obtained under controlled laboratory conditions. However, complex and dynamic environmental conditions, which plants actually face in nature, should be taken into account to model plant–microbe interactions. Given the significant impact of environmental factors on not only plants but also microbes, it will be a key future challenge to directly incorporate relevant information from a complex environment with changing conditions in order to understand the true nature of plant–microbe interactions. Considering recent technological advances and lowering cost for quantitative measurement such as RNA-seq, we propose that it is time to tackle the grand challenge with the power of systems biology.

## Conflict of Interest Statement

The authors declare that the research was conducted in the absence of any commercial or financial relationships that could be construed as a potential conflict of interest.
